# Type III Second Branchial Cleft Cyst: A Rare Presentation

**DOI:** 10.7759/cureus.28568

**Published:** 2022-08-30

**Authors:** Vaidehi Hande, Shraddha Jain, Chandra Veer Singh, Sana Parveen, Mithula Murali

**Affiliations:** 1 Otolaryngology, Jawaharlal Nehru Medical College, Datta Meghe Institute of Medical Sciences (Deemed to be University), Wardha, IND; 2 Otolaryngology - Head and Neck Surgery, Jawaharlal Nehru Medical College, Datta Meghe Institute of Medical Sciences (Deemed to be University), Wardha, IND; 3 Otolaryngology, Jawaharlal Nehru Medical College, Datta Meghe Institute of Medical Science (Deemed to be University), Wardha, IND

**Keywords:** neck swelling, neck mass, type iii, branchial cyst, branchial arch

## Abstract

The second branchial cleft cyst (BCC) is the most common type of BCC. Bailey proposed a classification of the second BCC into four types, among which a Bailey type II cyst is the most common presenting lateral to the carotid space. A Bailey type III cyst, which extends between internal and external carotid arteries is an extremely rare occurrence. Complete surgical excision is the treatment modality of choice for branchial cysts and this warrants thorough imaging to see the detailed extent of the cyst. Contrast-enhanced computed tomography and Magnetic resonance imaging, both aid in diagnosis. Here we report a rare case of Type III second BCC, which presented with dysphagia, along with an elaboration of findings on imaging and treatment details.

## Introduction

Major head and neck structures are created during the fifth week of fetal development. The five pharyngeal arches (tissue bands) are significant structures that develop. Several malformations or deformities in the neck originate from incomplete or failing, during the embryonic development of these arches. Cysts, sinuses, and fistulae are the three types of branchial arch abnormalities [1-3].

Sinuses showing as an aperture in the lower neck are the most prevalent presentation of branchial arch abnormalities. First branchial cleft anomalies present as recurrent painful swellings around the ear and sometimes may be associated with pus discharge from the sinus. In 1832, Ascherson coined the term “branchial cyst.” He hypothesized that these cysts were the result of poor branchial cleft obliteration [4]. Persistence of pre-cervical sinus, cystic degeneration of cervical lymph nodes, and thymo-pharyngeal ductal remnant are other proposed explanations of branchial cyst formation [4]. The most widely accepted idea to date is that the third and fourth arches, which are covered by the second arch, fail to involute during development, resulting in epithelial cells being trapped within and eventually developing into a branchial cyst.

We report a rare case of large Bailey type III second branchial cleft cyst (BCC) with an extension between internal and external carotid arteries and having overlapping features with type IV cyst, presenting with dysphagia as its deeper part formed relation with pyriform fossa, an occurrence which has probably not been reported previously.

## Case presentation

**Figure 1:**
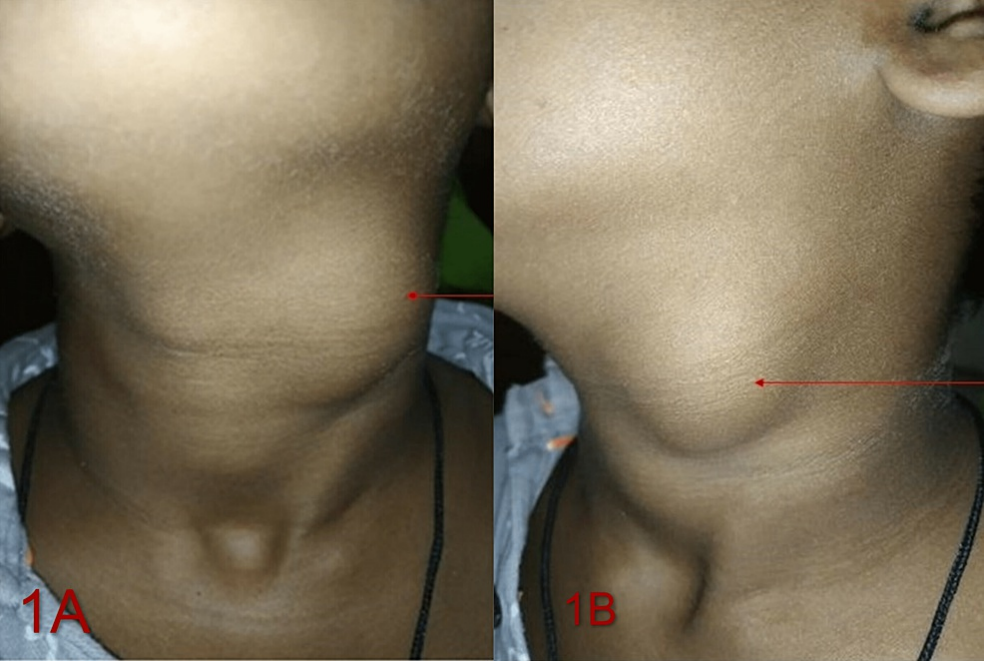
(A, B) Red arrow showing cyst over the upper antero-lateral aspect of neck.

An 11-year-old boy had been complaining for two months about swelling on the left side of his neck and difficulties swallowing. A 3 cm × 2.5 cm globular enlargement, anterior to the upper and middle part of the sternocleidomastoid, superiorly extending 2 cm below the ramus of mandible, 5 cm above the sternoclavicular joint, with smooth surface and well-defined edges, was present on clinical examination (Figures 1A, 1B). A non-tender, soft mass was palpated that was freely movable and not attached to any underlying structures. Based on the history nearest possible differential could have been cervical lymphadenitis, cervical metastasis, or occult primary.

**Figure 2:**
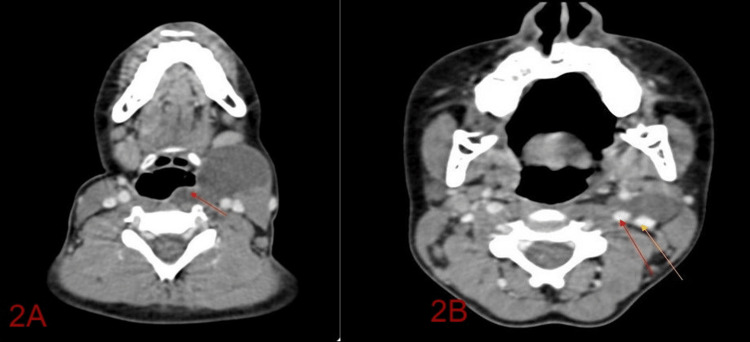
(A) Contrast-enhanced computed tomography showing cyst causing compression of the pyriform fossa (red arrow). (B) Contrast-enhanced computed tomography showing the cyst causing splaying of internal carotid arrow (red arrow) and external carotid arteries (yellow arrow).

A contrast-enhanced CT scan of the neck revealed a well-defined peripherally enhancing cystic mass measuring 4.7 x 2.9 x 4.8 cm, anterior to the sternocleidomastoid and extending medially up to the left lateral wall of the pharynx (Figure 2A). Splaying of the internal and external carotid due to a lesion that extended between them causing a bird beak appearance (Figure 2B).

**Figure 3:**
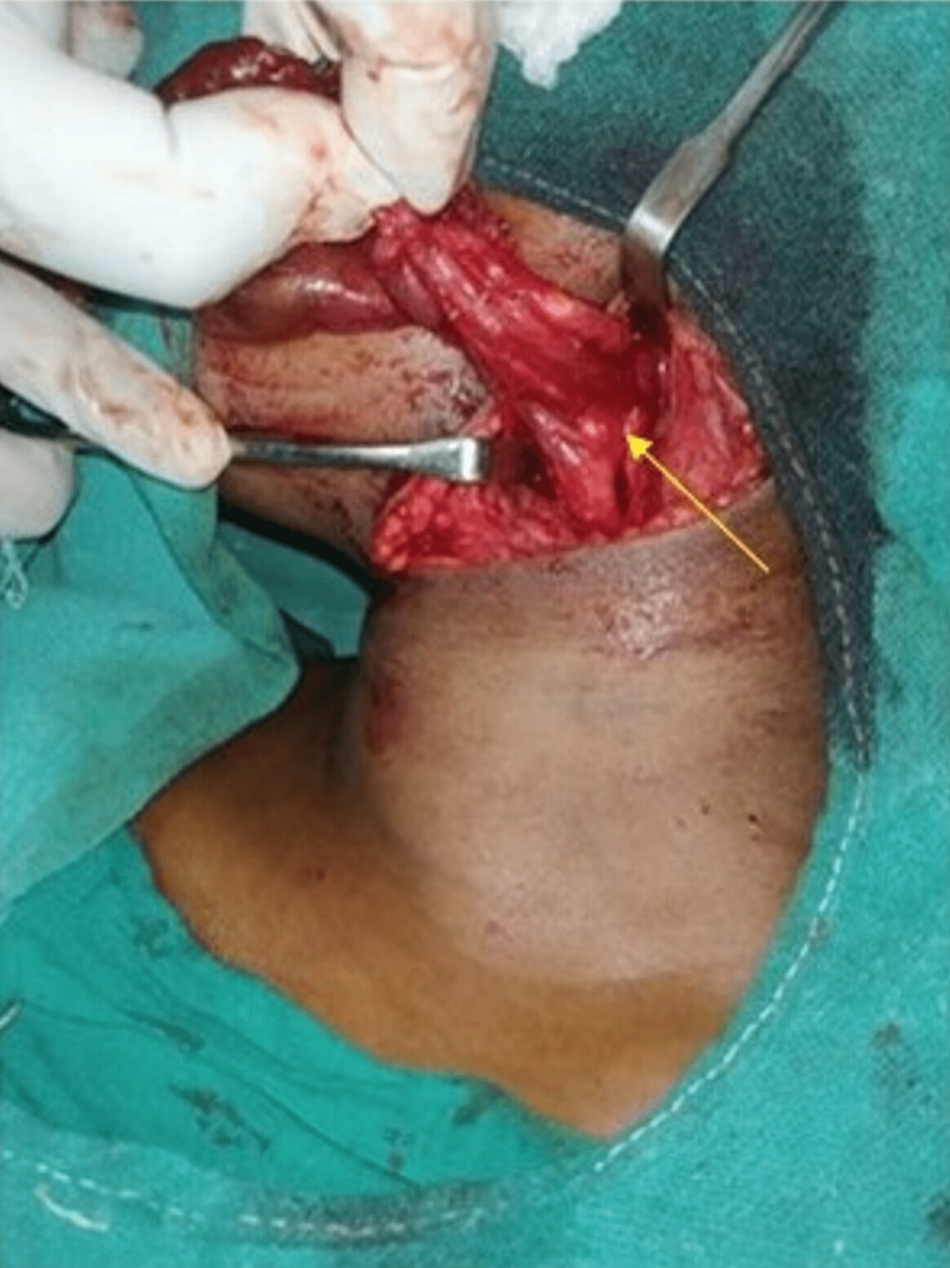
Intraoperative image showing cyst attached to the Internal carotid artery (yellow arrow).

The cyst was torn from its attachment, along with its tract that led to the pyriform fossa, and the patient had surgical excision. The cyst between the internal and external carotid arteries was meticulously dissected, with the glossopharyngeal nerve preserved (Figure 3). The post operative period was uneventful. No complications or scar site infections were encountered on a six-month follow up.

## Discussion

Bailey [5] classified second BCCs into four types. Type I cysts are located under the superficial cervical fascia at the anterior border of the sternocleidomastoid muscle. A type II cyst is classically placed lateral to the carotid space, and posterior to the submandibular gland and is the commonest type of second BCC. Type III cyst is known to have an extension between the bifurcation of the internal and external carotid arteries up to the lateral pharyngeal wall and is an extremely rare occurrence. The type IV cyst is situated medial to the carotid sheath in the pharyngeal mucosal space. A Bailey type III cyst is an uncommon occurrence.

Around 90%-95% of the cases are second branchial cleft abnormalities, which usually manifest clinically between the ages of 20 and 40 [4,6-8]. We report a second BCC in an 11-year-old child. The swelling presented in the neck as a cystic lateral neck swelling, located in the mid-neck, which was much higher than the classic location of the second branchial arch cyst, at the junction of the middle and lower one-third of the neck. About 20% of cervical lesions in children are BCCs and fistulae [8]. In the differential diagnosis of head and neck masses in children and adults, congenital cervical abnormalities must be considered. These lesions might be felt as cystic masses, infectious masses, or fistulae [8].

Anywhere along the route that passes posterior to the carotid arteries, pierces the thyrohyoid membrane, penetrates the larynx, and ultimately terminates on the lateral face of the pyriform sinus is where BCCs can be detected. To differentiate between the two, consider the superior laryngeal nerve's relationship to the sinus tract. The third arch sinuses start at the base of the pyriform sinus and run posteriorly to the carotid artery, passing superior to the glossopharyngeal nerve but inferior to the superior laryngeal nerve and the hypoglossal nerve. From the top of the pyriform sinus, the fourth arch sinuses continue inferior to the superior laryngeal nerve and descend the tracheoesophageal groove [9]. The second arch cyst is the most frequent [10]. Structural malformations of the auricle may also be associated [11]. Cervical lymphadenitis and tuberculous lymphadenitis, infection in the neck spaces, infectious mononucleosis, cat-scratch disease and syphilis are some of the causes presenting as infectious masses in the neck [12].

On computed tomography, they appear as fluid-attenuated masses with distinctive edges. When proteinaceous debris is present, magnetic resonance imaging exhibits varying cyst wall thickness and contents that may be either hypo- or hyperintense to muscle. BCCs are rather intense on T2-weighted scans.

The therapy of choice is surgical excision, which has a favorable prognosis. The diagnosis of a third BCC was verified by a histopathologic study of the tumor removed from our patient. The inflammatory squamous mucosa and surrounding lymphoid tissue are typical BCC pathologic features.

## Conclusions

Second branchial cysts exhibit a variety of sonographic appearances that can be confusing to the untrained eye. For an appropriate diagnosis, the clinician performing the investigation must be well-versed in this area. There is no gender predilection or proclivity for the position of branchial abnormalities (right or left). The overlapping features of type III and IV Bailey Classification for type 2 BCC were found in our case. The use of imaging in the identification and treatment of the lesion was critical. The pre-operative knowledge of the extent of the cyst facilitates complete surgical clearance.
